# Sex hormone deficiency in male and female mice expressing the Alzheimer’s disease-associated risk-factor TREM2 R47H variant impacts the musculoskeletal system in a sex- and genotype-dependent manner

**DOI:** 10.1093/jbmrpl/ziae144

**Published:** 2024-11-13

**Authors:** Roquelina Pianeta, Padmini Deosthale, Natasha Sanz, Rachel Kohler, Chiebuka Okpara, Matthew Arnett, Iqra Asad, Amber Rogers, Madison Gerbig, Alyson Essex, Ziyue Liu, Joseph M Wallace, Lilian I Plotkin

**Affiliations:** Department of Anatomy, Cell Biology & Physiology, Indiana University School of Medicine, Indianapolis, IN 46202, United States; Indiana Center for Musculoskeletal Health, Indiana University School of Medicine, Indianapolis, IN 46202, United States; Richard L. Roudebush Veterans Administration Medical Center, Indianapolis, IN 46202, United States; Department of Anatomy, Cell Biology & Physiology, Indiana University School of Medicine, Indianapolis, IN 46202, United States; Richard L. Roudebush Veterans Administration Medical Center, Indianapolis, IN 46202, United States; Department of Anatomy, Cell Biology & Physiology, Indiana University School of Medicine, Indianapolis, IN 46202, United States; Bone Biology Laboratory, School of Medicine, Rosario National University, Rosario 2000, Argentina; National Council of Scientific and Technical Research (CONICET), Buenos Aires 9100, Argentina; Department of Biostatistics and Health Data Science, Indiana University School of Medicine, Indianapolis, IN 46202, United States; Department of Anatomy, Cell Biology & Physiology, Indiana University School of Medicine, Indianapolis, IN 46202, United States; Department of Anatomy, Cell Biology & Physiology, Indiana University School of Medicine, Indianapolis, IN 46202, United States; Department of Anatomy, Cell Biology & Physiology, Indiana University School of Medicine, Indianapolis, IN 46202, United States; Department of Anatomy, Cell Biology & Physiology, Indiana University School of Medicine, Indianapolis, IN 46202, United States; Department of Anatomy, Cell Biology & Physiology, Indiana University School of Medicine, Indianapolis, IN 46202, United States; Department of Anatomy, Cell Biology & Physiology, Indiana University School of Medicine, Indianapolis, IN 46202, United States; Indiana Center for Musculoskeletal Health, Indiana University School of Medicine, Indianapolis, IN 46202, United States; Indiana Center for Musculoskeletal Health, Indiana University School of Medicine, Indianapolis, IN 46202, United States; Department of Biostatistics and Health Data Science, Indiana University School of Medicine, Indianapolis, IN 46202, United States; Indiana Center for Musculoskeletal Health, Indiana University School of Medicine, Indianapolis, IN 46202, United States; Richard L. Roudebush Veterans Administration Medical Center, Indianapolis, IN 46202, United States; Department of Biomechanical Engineering, Indiana University—Purdue University Indianapolis, Indianapolis, IN 46202, United States; Department of Anatomy, Cell Biology & Physiology, Indiana University School of Medicine, Indianapolis, IN 46202, United States; Indiana Center for Musculoskeletal Health, Indiana University School of Medicine, Indianapolis, IN 46202, United States; Richard L. Roudebush Veterans Administration Medical Center, Indianapolis, IN 46202, United States

**Keywords:** bone mass and strength, gonadal steroid hormones, sexual dimorphism, TREM2, skeletal muscle

## Abstract

The R47H variant of the triggering receptor expressed on myeloid cells 2 (TREM2) is a risk factor for Alzheimer’s disease in humans and leads to lower bone mass accrual in female but not male 12-mo-old mice. To determine whether, as with aging, gonadectomy results in sex-specific musculoskeletal effects, gonad removal or SHAM surgery was performed in 4-mo-old TREM2^R47H/+^ mice and WT male and female littermates (*n* = 10-12/group), with sexes analyzed separately. Body weight was lower in males, but higher in females after gonadectomy, independently of their genotype. Gonadectomy also leads to decreased BMD in males at all sites and in the whole body (total) and spine in female mice for both genotypes. Total and femur BMD was lower in gonadectomized male mice 6-wk post-surgery, independently of the genotype. On the other hand, BMD was only lower in ovariectomized WT but not TREM2^R47H/+^ mice in all sites measured at this time point. Bone formation and resorption marker levels were not affected by orchiectomy, whereas CTX was higher 3 wk after surgery and P1NP showed a tendency toward lower values at the 6-wk time point only in ovariectomized WT mice. Micro-CT analyses showed no differences resulting from gonadectomy in structural parameters in femoral cortical bone for either sex, but lower tissue mineral density in males of either genotype 6-wk post-surgery. Nevertheless, biomechanical properties were overall lower in gonadectomized males of either genotype, and only for WT ovariectomized mice. Distal femur cancellous bone structure was also affected by gonadectomy in a genotype- and sex-dependent manner, with genotype-independent changes in males, and only in WT female mice. Thus, expression of the TREM2 R47H variant minimally alters the impact of gonadectomy in the musculoskeletal system in males, whereas it partially ameliorates the consequences of ovariectomy in female mice.

## Introduction

Alzheimer’s disease (AD) is the most common form of dementia and remains as the fifth cause of death among population over 65 yr old.^1^ Alzheimer’s disease is a neurodegenerative condition, characterized by an impairment of vital processes such as cognitive functions and speech, followed by compromising activities such as mastication and walking, due to progressive damage of the neurons.^1^ Clinical and preclinical data support the hypothesis that involves the abnormal accumulation of two proteins, amyloid beta (Aβ) and hyperphosphorylated tau, as the main cause of neuron destruction and irreversible damage of the synaptic connections, possibly due to malfunction of the microglial cells in the brain.^2^^,^^3^ Microglia are specialized immune cells that reside in the central nervous system, and are responsible for Aβ clearance and synapsis maintenance.^4^ Microglia, as osteoclasts, derive from the hematopoietic stem cells and both cell types share the expression of the triggering receptor expressed on myeloid cells 2 (TREM2).^4^ The triggering receptor expressed on myeloid cells 2 participates in the phagocytic activity of the microglia^4^^,^^5^ and the differentiation of osteoclasts.^4^^,^^6^^,^^7^ A TREM2 SNP, the R47H variant in which arginine 47 is replaced by an histidine (p.R47H, rs75932628) in the extracellular immunoglobulin domain, has been linked to a 3-4-fold increased risk of developing AD.^8^^,^^9^ Moreover, the TREM2 R47H variant expression accelerates the path from preosteoclasts to mature osteoclasts.^4^

Clinical data suggest a connection between declined cognitive function and reduced bone quality.^10^ Although both microglia and osteoclasts participate in AD and osteoporosis, respectively, and the TREM2 R47H variant affects the functions of both cell types, understanding the link between this SNP and the two diseases affecting different systems remains challenging.

In a previous study, we characterized the phenotype of mice expressing one copy of the TREM2 R47H variant (TREM2^R47H/+^), from a musculoskeletal perspective.^11^ Our findings suggest that AD-associated TREM2 R47H variant impacts the musculoskeletal system in a sexual-dimorphic manner independent of central neuropathology.

Epidemiological data regarding the relationship among sex, musculoskeletal decline, and AD are still unclear, although several physiological and cellular mechanisms have been proposed to explain the potential common pathways and risk factors.^12^ In particular for women, pregnancy and menopause are considered to confer increased risk to develop AD.^13^ Also, clinical presentation and early signs related to AD development differ between women and men.^13^ In a similar fashion, bone-related diseases such as osteoporosis differ between women and men, and the role of sex hormones on the bone remodeling process is a key factor to understand such differences.^14^ Estrogens and androgens control osteoclastogenesis by affecting the energy metabolism of preosteoclasts and modulating their apoptosis.^15^^,^^16^ Estrogen production is compromised in women reaching menopause, increasing the risk of developing osteoporosis, whereas there is not a physiological andropause in men but a decline in androgen production with aging.^16^^,^^17^ It is well known that androgens are necessary for muscle anabolism, and physical impairment is expected in older men.^17^ Although reduced physical activity has been linked to a higher risk of AD development, the mechanisms behind such connection may differ between aged women and men.^12^

Mice do not develop menopause naturally, but experimentally-induced menopause in sexually mature female mice results in cognitive decline.^18^ TREM2^R47H/+^ females exhibit higher muscle strength with age, in contrast to male mice, when compared to their WT littermates.^11^ However, bone phenotype of both female and male mice did not differ between WT and mutant mice at 4 mo of age. Thus, to determine the impact of estrogens and androgens on the musculoskeletal phenotype of mice expressing the TREM R47H variant, here we use the surgical remotion of the gonads (gonadectomy)^19^ in mice at 4 mo of age, to induce sex hormone deficiency.

## Materials and methods

### Mice

**Figure 1:**
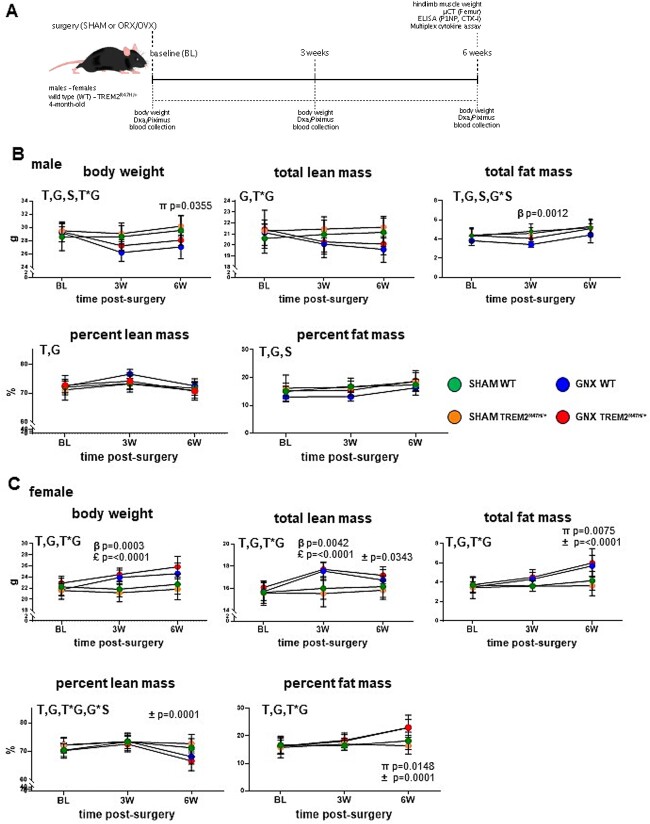
Gonadectomy alters body weight and composition for each sex partially in a genotype-dependent manner. (A) Schematic representation of timeline for surgery and analyses of mice reported in this study. (B, C) Body weight and composition measured by DXA/PIXImus before (baseline) and at the indicated times after gonadectomy in male (B) and female (C) WT or TREM2^R47H/+^ mice. Each dot corresponds to the mean of the group, and the vertical lines are SDs. Data were analyzed by 3-way ANOVA, followed by Tukey’s post-hoc test. Symbols indicate *p* values for the post-hoc tests: β = WT: 3W-SHAM vs 3W-GNX; π = WT: 6W-SHAM vs 6W-GNX; £ = TREM2^R47H/+^: 3W-SHAM vs 3W-GNX; **±** = TREM2^R47H/+^: 6W-SHAM vs 6W-GNX. *p* values for all other comparisons are >.05. Significant overall effects are indicated by the following letters: T = time post-surgery effect, S = surgery effect, G = gonadectomy effect, T^*^G = time post-surgery × genotype interaction; G^*^S = genotype × surgery interaction. *N* = 9-10/group. Abbreviation: TREM2, triggering receptor expressed on myeloid cells 2.

Male and female C57BL/6J mice expressing the R47H TREM2 variant (TREM2^R47H/+^ mice) and their WT littermates (wild type; TREM2^+/+^), used as controls, were generated as previously published.^11^ To determine the presence of the mutation, and its heterozygous status, genotyping for SNP was performed based on corresponding primers (*forward: 5′-ATGTACTTATGACGCCTTGAAGCA* and *reverse: 5′-ACCCAGCTGCCGACAC*) and SNP reporters (*forward: 5′-CCTTGCGTCTCCC* and *reverse: 5′-CCTTGTGTCTCCC*).^11^ All animals were maintained under standardized conditions (5 mice/cage, diet and water *ad libitum*; 12-hr light/dark cycle). Following genotyping, mice were randomly assigned to the SHAM or gonadectomy group. All procedures (summarized in Figure 1A) were approved by the Institutional Animal Care and Use Committee of Indiana University School of Medicine.

### Sex hormone deficiency mouse model

Male and female WT and TREM2^R47H/+^ mice underwent orchiectomy or ovariectomy, respectively, at 4 mo of age (Figure 1A). All mice received pre-operative analgesia subcutaneously and were operated under general isoflurane anesthesia, connected to a nose cone. Previous induction in isoflurane chamber was performed per protocol. Before incisions, the surgical area was shaved and disinfected to ensure aseptic conditions. For male mice, supine position was secured with limbs and tail fixation. Then, a single ventral suprapubic skin incision was performed, and fat pad and the testis from each side were removed.^19^ For female mice, the remotion of the ovaries was performed using double dorsolateral incisions while fixed in prone position.^19^^,^^20^ Hot tip holder hemostasis was achieved after gonad removal, and muscle suture combined with wound clips for skin incision were used for both sexes. SHAM mice underwent the same surgical procedure, except for gonad removal (each organ was verified and carefully manipulated but remained in situ). All mice recovered assisted with heat pads (indirect contact) and were followed during 7 d before wound clip removal. Ovariectomy efficacy was verified with uterus atrophy. All mice were euthanized at 6-wk post-surgery, using cervical dislocation, confirmed by chest auscultation. The hindlimb muscles and the uteri were harvested and weighted. In addition, the left femur was collected, cleaned of soft tissue, and stored at −20 °C until further processing for micro-CT (μCT) and mechanical testing, and the right femur was fixed in 10% neutral buffered formalin for histomorphometry. Ten to 12 mice were included per group, and all animals survived until the end of the experiments without complications.

### Body weight, body composition, and BMD

For all mice, body weight, BMD for total (except head and tail), spine (L1-L6), and femur, and total body lean and fat mass, were measured at baseline (ie, before surgery), and at 3- and 6-wk post-surgery, using a DXA/PIXImus (GE Medical Systems, Lunar Division, Madison, WI, USA).^11^ The device was calibrated according to the manufacturer instructions, using a standardized phantom before each imaging procedure.

### Bone microstructure with X-ray μCT

Prior to bending tests, left femurs were thawed, wrapped in parafilm, and scanned in groups of 3 in a Bruker Skyscan 1272 at isotropic 10 μm voxel size (70 kV, 142 μA, 0.7-degree angle step, 2 frames averaged).^21^^,^^22^ Scans were then reconstructed (NRecon) and rotated (DataViewer) before calibrating to hydroxyapatite-mimicking phantoms (0.3 and 1.25 g/cm^3^ CaHA). Bone was segmented from non-bone using a grayscale threshold of 70. Trabecular ROIs were chosen as 1 mm extending proximally from the proximal edge of the distal growth plate, and cancellous architecture in each ROI was quantified using custom processing functions in CT Analyzer (CTAn). Cortical properties were measured within a 0.1-mm cortical ROI taken from the central midshaft (approximately 50% of femoral length) then analyzed with a custom MATLAB program (MathWorks, Inc. Natick, MA). After scans, bones were re-wrapped in PBS-soaked gauze and stored at −20 °C until bending tests were performed.

### Mechanical testing (3-point bending)

For 3-point bending analysis, left femurs were removed from −20 °C storage and placed in a warm room (37 °C) for 1 hr to thaw prior to bending tests. Each femur was tested to failure in 3-point bending (support span at 8.7 mm), with the anterior surface in tension (TA Instruments ElectroForce 5500). Bones were loaded at a displacement control rate of 0.025 mm/s while the sample remained hydrated with PBS. Force and displacement values were recorded. Cross-sectional cortical properties for each bone were obtained by re-analyzing a 0.5-mm ROI of μCT slices from around the fracture site, as described above. Another custom MATLAB program was used to construct a force-displacement curve and normalize it with μCT data and standard engineering bending equations to produce a stress–strain curve. Yield was identified using the 0.2% strain offset method. Force-displacement data were used to determine yield force, maximum force, displacement at yield, post-yield displacement, and total displacement. Stiffness was taken from the linear slope in the elastic region and work to yield, post-yield work, and total work were calculated from the area underneath the curve. Stress–strain data were used to determine yield stress, ultimate stress, strain to yield, and total strain. The slope of the stress–strain curve in the elastic region was taken as the elastic modulus, the area under the elastic region of the curve was taken as the resilience, and the area under the entire curve was taken as toughness.^23^

### Bone histomorphometry

Right femora were processed by standardized methods at the Indiana Center for Musculoskeletal Health Histology and Histomorphometry laboratory.^24^ For dynamic histomorphometry, all mice were injected with calcein (30 mg/kg; Sigma-Aldrich, St. Louis, MO, USA) and alizarin red (50 mg/kg; Sigma-Aldrich), intraperitoneally, 7 and 2 d before euthanasia, respectively. Then, un-demineralized, MMA-embedded cortical (mid-diaphysis) cross-sections or cancellous (distal femur) longitudinal sections were analyzed using epifluorescence microscopy. Subsequent slides were stained for von Kossa/McNeal and analyzed under bright field for static histomorphometry, as reported.^25^ All analyses were performed using an OsteoMeasure high-resolution digital video system (OsteoMetrics Inc., Decatur, GA, USA), and abbreviations and units are reported following standardized guidelines.^26^

### Serum measurements

Blood samples were obtained from all mice at the 3-wk and 6-wk time points, after 3 hr of fasting. For each sample, serum was separated and stored at −80 °C until processing. Plasma N-terminal P1NP (cat.#AC-33F1) and C-telopeptide fragments (CTX-I) (cat.#AC-06F1) were detected using ELISA kits from Immunodiagnostic Systems Inc. kits (Fountain Hill, AZ, USA), following the manufacturer’s instructions.

The levels of circulating cytokine/chemokines were measured in serum obtained 3-wk post-surgery, using the Milliplex mouse cytokine/chemokine 32 plex kit (Milliplex, Burlington, MA). Data are reported as pg/mL of whole serum.

### Statistical analysis

Results are reported as minimum to maximum values with median and IQR (25th to 75th percentile), showing individual data points in all figures. Values at baseline were compared non-paired by *t*-test. Linear regressions models were adopted instead of 2-way ANOVA when comparing 4 groups within each sex, because of the unequal sample sizes among groups. Both genotype and surgery were included as categorical variables with their interaction terms. For outcomes that the overall model showed statistically significant, post-hoc pairwise comparisons were conducted for (1) R47H/+ GNX vs R47H/+ SHAM, (2) WT GNX vs WT SHAM, (3) R47H/+ GNX vs WT GNX, and (4) R47H/+ SHAM vs WT SHAM (male and female separately). Bonferroni corrections were applied to the *p*-values and the 95% simultaneous CIs. Two-sided *p*-values <.05 were considered as statistically significant. Time courses were analyzed by 3-way ANOVA with time after surgery, sex, and genotype as the independent variables, followed by Tukey’s multiple comparisons test. All analyses were performed using SAS 9.4 (SAS Institute, Cary, NC, USA) or GraphPad (GraphPad Software, San Diego, CA, USA). The results of the statistical analyses are shown in the figures and in Tables S1 and S2, with differences with *p* < .05 highlighted in yellow and those between 0.05 and 0.06 in light blue.

## Results

### Sex hormone deficiency leads to genotype- and sex-dependent changes in body weight and body composition

No differences between WT and TREM2^R47H/+^ mice were found at baseline in body weight or composition in males or females (Figure 1B and C), whereas all measurements showed overall significant time post-surgery and genotype effects for males and females (except for time post-surgery, which did not alter significantly total lean mass in males). In addition, there was a significant surgery effect for body weight and fat mass (total and percent of body weight) for males, with significant time post-surgery × genotype interaction for body weight and genotype × surgery interaction for total fat mass (Figure 1B). Post-hoc analyses showed only minor differences, with lower body weight and fat mass for orchiectomized WT mice compared to SHAM littermates at 6- and transiently 3-wk post-surgery, respectively. Further, body weight was only different for gonadectomized WT male mice, with lower values 3 wk after orchiectomy compared to the same animals at baseline (Table S1). No differences within the time of the experiment were seen in body weight for mutant mice or in body composition for mice of either genotype or surgery.

In summary, there is no change in weight in mice of either genotype (Figure 1B and Table S1) following orchiectomy. However, at the end of 6 wk, only WT mice have significantly less weight compared to SHAM-operated littermates. This change in weight is not associated to significant changes in lean mass or fat mass, with the exception of a significant increase in fat mass at 3 wk in WT mice, not seen at 6-wk post-surgery.

More differences were detected for female mice, with time post-surgery × genotype interactions for all measurements, and an additional genotype × surgery interaction for percent lean mass (Figure 1C). Post-hoc analyses showed significantly higher body weight and lean mass (but not percent lean mass) for ovariectomized mice independently of the genotype, compared to the respective SHAM mice at the 3-wk time point. By 6-wk post-surgery, only ovariectomized TREM2^R47H/+^ mice maintained higher lean mass, and lower percent lean mass, whereas percent fat mass was higher for ovariectomized mice of either genotype. Comparisons between time points showed gain in body weight after ovariectomy at both time points for WT mice and 6-wk post-surgery for mutant mice compared to the respective baseline (Table S1). Total lean mass was higher at the 3-wk time point for females of either genotype, whereas the percent lean mass decreased at the last time point only for WT mice that underwent ovariectomy, compared to baseline values. Fat mass in grams and as percent of body weight was higher for mice of both genotypes when the mice were compared between baseline and 6-wk post-ovariectomy. No changes were observed in male or female SHAM-operated mice at any time point. Further, no differences were detected between WT and TREM2^R47H/+^ mice either SHAM or ovariectomized at any time point (all *p* values >.05).

Therefore, both genotypes show a significant increase in weight post-ovariectomy with no difference between genotypes at 6 wk after surgery. Further, only ovariectomized mutant mice have lower percent lean mass compared to SHAM animals of the same genotype, whereas fat mass (both total and percent) was similarly affected in WT and TREM2^R47H/+^ mice 6-wk post-ovariectomy. These results suggest a differential consequence of the mutation in lean versus fat mass following gonadectomy in female mice.

At the time of euthanasia, only minor changes were detected in skeletal muscle weight (Figure S1). Thus, although there was a significant effect of the surgeries on tibialis anterior, gastrocnemius, and quadriceps muscles, only tibialis anterior and quadriceps from gonadectomized male TREM2^R47H/+^ had lower weight compared to SHAM littermates of the same genotype. Further, there was an overall model effect for quadriceps weights in males and females, and a significant genotype effect with lower weight in orchiectomized TREM2^R47H/+^ mice compared to SHAM mice of the same genotype.

Therefore, the consequences of sex steroid removal on weight, lean/fat, and muscle mass depend on the genotype of the mice with a partial contribution of the gonadectomy over the course of the experiment. Further, the effects of the surgeries are more evident in females compared to male mice, independently of the genotype for whole body measurements. On the other hand, skeletal muscle weight (tibialis anterior and quadriceps) was only affected in male mutant mice at the end of the experiment.

### Gonadectomy results in similar loss of bone mass in WT and TREM2^R47H/+^ male mice, whereas it only leads to low bone mass in WT females, compared to SHAM-operated mice 6-wk post-surgery

At baseline, total and femur BMD were slightly but significantly higher in male TREM2^R47H/+^ compared to WT littermates (0.052 ± 0.002 vs 0.053 ± 0.002 g/cm^2^ for total BMD and 0.073 ± 0.004 vs 0.076 ± 0.003 g/cm^2^ for femur BMD of WT and TREM2^R47H/+^, respectively). On the other hand, no difference was seen in spine BMD (0.057 ± 0.003 vs 0.058 ± 0.003 g/cm^2^ for WT and TREM2^R47H/+^, respectively) in the males or in any sites in the females. These genotype-dependent differences were not seen when SHAM animals of either sex were compared 3 and 6 wk after surgery.

**Figure 2:**
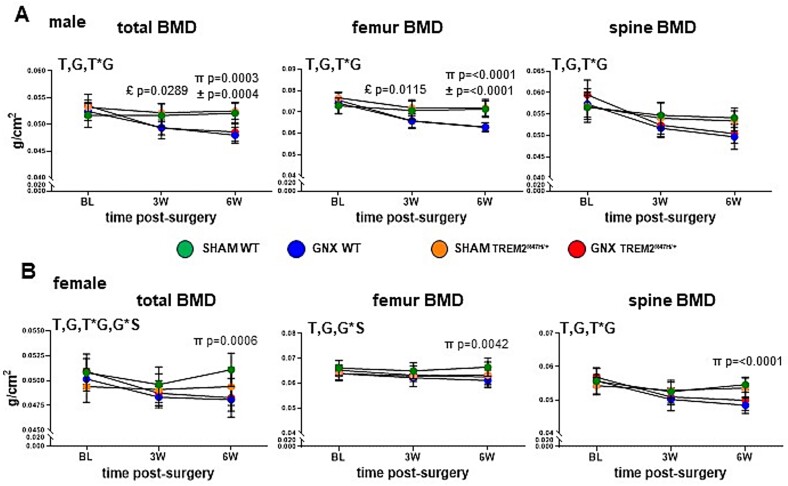
Bone loss following sex steroid removal depends on the genotype for female but not for male mice. (A, B) BMD measured in vivo by DXA/PIXImus at the time of the surgery, and 3 and 6 wk following gonadectomy. Analyses were performed for total (minus head and tail), lumbar spine, and femur for male (A) and female (B) mice. Each dot corresponds to the mean of the group, and the vertical lines are SDs. Data were analyzed by 3-way ANOVA, followed by Tukey’s post-hoc test. Symbols indicate *p* values for the post-hoc tests: π = WT: 6W-SHAM vs 6W-GNX, £ = TREM2^R47H/+^: 3W-SHAM vs 3W-GNX, **±** = TREM2^R47H/+^: 6W-SHAM vs 6W-GNX. *p* values for all other comparisons are >.05. Significant overall effects are indicated by the following letters: T = time post-surgery effect, S = surgery effect, G = gonadectomy effect, T^*^G = time post-surgery × genotype interaction; G^*^S = genotype × surgery interaction. *N* =10-11/group. Abbreviation: TREM2, triggering receptor expressed on myeloid cells 2.

Three-way ANOVA analyses revealed significant time post-surgery and genotype effects, and interaction of the 2 variables for male mice in all sites measured. Comparison of BMD between baseline and 3- or 6-wk post-surgery showed decline at both time points independently of the genotype (Table S1). Total and femur BMD were lower for the mutant, but not WT mice, 3-wk post-surgery, and for both genotypes at the terminal BMD in both sites compared to SHAM-operated mice at each time point, suggesting an accelerated loss of bone mass in the orchiectomized TREM2^R47H/+^ mice (Figure 2A). No differences were detected in spine BMD between any 2 groups at any time point.

For females, in addition to the time post-surgery and genotype overall effects seen at all sites, there was a significant time post-surgery × genotype interaction for total and spine BMD, and a genotype × surgery interaction for total and femur BMD (Figure 2B). The analyses of the time courses showed that female TREM2^R47H/+^ subjected to gonadectomy loss total and spine, but not femur BMD within the course of the experiment, similar to gonadectomized WT littermates (Table S1). However, when SHAM and ovariectomized mice were compared, only gonadectomized WT mice had lower BMD compared to SHAM-operated female mice of the same genotype 6 wk after surgery, with no other differences at baseline or 3-wk post-surgery. Taking together, this evidence suggests that although female TREM2^R47H/+^ mice lose bone with ovariectomy, they appear to be less susceptible to the consequences of the surgery than WT littermates.

There was an overall effect of time post-surgery for both P1NP and CTX levels in males and females, whereas genotype only influenced CTX levels in animals of both sexes (Figure S2). In addition, the statistical analyses showed interactions between time post-surgery and genotype and among time post-surgery, genotype, and surgery for P1NP levels, and an interactions between time post-surgery and genotype for CTX in females. Post-hoc analyses showed only a significant difference between 3- and 6-wk post-surgery for CTX levels in gonadectomized WT, but not mutant females (Table S1). Further, CTX levels were higher in ovariectomized WT mice at the 3-wk time point, with a tendency toward lower P1NP levels in gonadectomized female WT mice 6-wk post-surgery (Figure S2).

Overall, our data suggest expression of the TREM2 R47H variant results in resistance to the effects of ovariectomy on BMD in female mice, compared to WT littermates, whereas it appears to accelerate bone loss induced by orchiectomy in male mice. Thus, the rate of loss of BMD post-gonadectomy is worse in male mutant compared to WT mice. On the other hand, while there is not an overall effect of the ovariectomy in either genotype, there is a genotype × surgery interaction in female mice that only renders significant differences in total and femur bone mass in WT mice. Therefore, our data suggest that the presence of the mutation has opposite consequences in the response of the skeleton to gonadectomy in males versus females.

### Expression of the TREM2 R47H variant partially prevents the effect of gonadectomy on cortical bone strength in female mice

**Figure 3:**
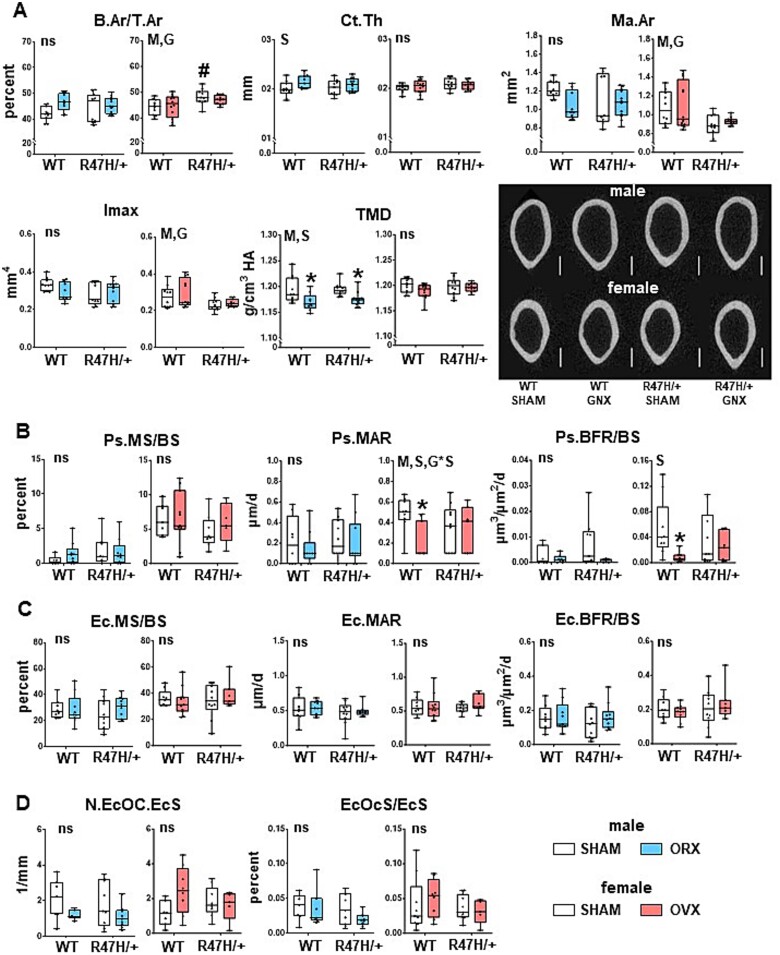
Gonadectomy leads to changes in cortical bone in a sex- and genotype-dependent manner. (A) Relative bone area (B.Ar/T.Ar), cortical thickness (Ct.Th), marrow cavity area (Ma.Ar), and maximum moment of inertia (Imax) and tissue mineral density (TMD) were measured in the femoral mid-diaphysis of 5.5-mo-old mice, 6-wk post-surgery. *N* = 9-11/group. Representative images of the μCT scans. Scale bar corresponds to 500 μm. (B, C) Dynamic histomorphometric analyses were performed on the periosteal (B) and endocortical (C) surfaces of cross-sections of the femoral mid-diaphysis. (D) Osteoclasts were quantified on the endocortical surface close to the femoral mid-diaphysis. *N* = 6-10/group. Minimum to maximum values with median and IQR (25th to 75th percentile) are shown, and each dot corresponds to an individual sample. ^*^ = *p*<.05 vs corresponding SHAM, determined using linear regression models. M = overall model significant effect, S = surgery effect, G = genotype effect, and G^*^S = genotype × surgery interaction. Abbreviations: μCT, micro-CT; IQR, interquartile range.

Micro-CT and histomorphometric analyses showed that cortical bone at the femoral mid-diaphysis was largely unchanged by surgery or genotype (Figure 3). The only exceptions were lower volumetric tissue mineral density in orchiectomized mice of either genotype, and periosteal mineral apposition and bone formation rate in WT gonadectomized 5.5-mo-old female mice, the age at the end of the experiment (Figure 3A). We also found higher relative bone area in SHAM, but not ovariectomized TREM2^R47H/+^ females, compared to SHAM-operated WT littermates. Of note, the differences in this bone compartment do not follow those seen in BMD (Figure 2).

**Figure 4:**
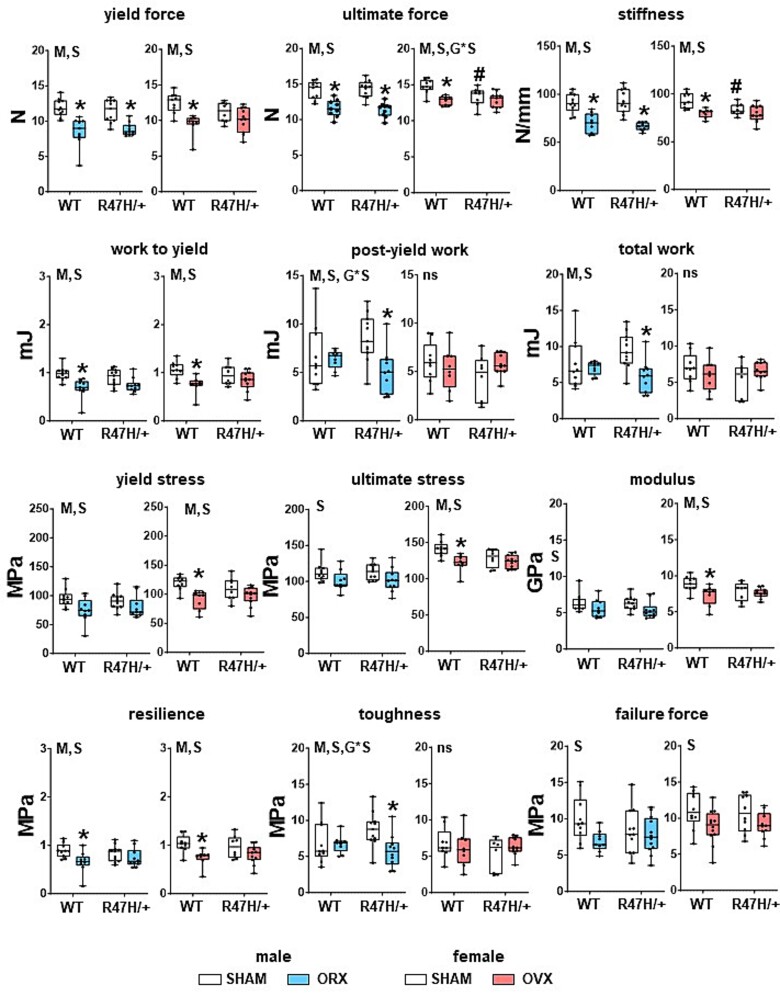
Biomechanical properties are altered following gonadectomy in a genotype- and sex-dependent manner. Biomechanical properties measured by 3-point bending assays in femur. Minimum to maximum values with median and IQR (25th to 75th percentile) are shown, and each dot corresponds to an individual sample. ^*^ = *p*<.05 vs corresponding SHAM and # = *p*<.05 vs WT mice with the same surgery, determined using linear regression models. M = overall model significant effect, S = surgery effect, G = genotype effect, and G^*^S = genotype × surgery interaction. *N* = 9-11/group. Abbreviation: IQR, interquartile range.

In spite of the lack effects at the structural levels, both surgery and genotype, and their interaction, affected the outcomes of the biomechanical testing (Figure 4). Thus, whereas no differences were seen between SHAM-operated males of the two genotypes, ultimate force and stiffness, but no other parameters, were lower in SHAM-TREM2^R47H/+^ female mice compared to WT littermates. Regarding the effects of the surgery, most mechanical properties showed overall significant effects for males (model, M, *p*<.05, Table S2) and were lower after gonadectomy for both WT and TREM2^R47H/+^ mice, compared to the corresponding SHAM-operated mice, with the exception work-related measurements. On the other hand, the only measurements that showed surgery × genotype interactions were post-yield work, and toughness, with tendencies toward significance in total work and yield stress for males, and significant difference in ultimate force and a tendency toward significance for ultimate stress for females (Table S2).

**Figure 5:**
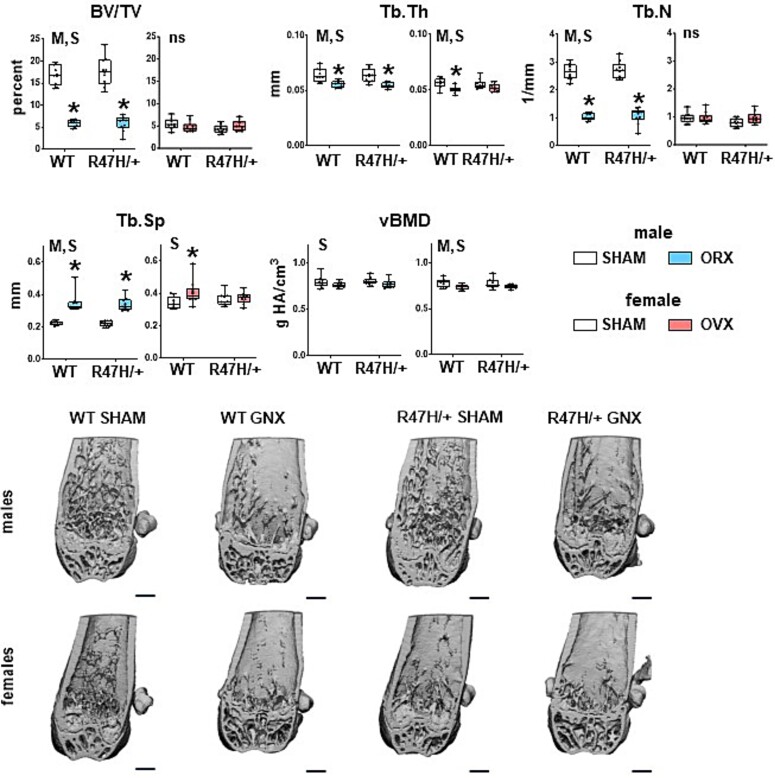
Gonadectomy results in lower cancellous bone mass in males independently of the genotype, but affects only WT, and not mutant, female mice. Bone structural parameters of the cancellous bone of distal femur measured by μCT. Minimum to maximum values with median and IQR (25th to 75th percentile) are shown, and each dot corresponds to an individual sample. ^*^ = *p*<.05 vs corresponding SHAM, determined using linear regression models. M = overall model significant effect, S = surgery effect, and G = genotype effect. *N* = 8-11/group. Representative μCT images. Scale bar corresponds to 500 μm. Abbreviations: μCT, micro-CT; IQR, interquartile range.

Post-hoc analyses showed that pre-yield-related parameters were lower only for gonadectomized male WT mice (work to yield and resilience), with a tendency toward lower values in yield stress (*p*=.05757, Table S2), whereas post-yield-related parameters were lower for orchiectomized mutant mice (post-yield and total work and toughness), compared to the respective SHAM controls. No differences were detected for ultimate strain or displacement yield for any comparisons (not shown), with only a tendency toward genotype × surgery interaction for displacement to yield (*p*=.06207, Table S2).

On the other hand, ovariectomy resulted in lower levels of parameters associated with both mechanical and material properties only for WT mice, with no differences detected in TREM2^R47H/+^ in any of the parameters measured (Figure 4 and Table S2). We also detected a reduction in ultimate force and stiffness for SHAM mutant female mice compared to SHAM WT mice, not seen in the previous study in 4-mo-old mice,^11^ suggesting that the deterioration on bone strength seen in 13-mo-old female mice starts to occur at approximately 5.5 mo of age. Further evidence of bone deterioration at this age (6-wk post-surgery) in female mice is shown by the levels of P1NP in the circulation, lower in SHAM-TREM2^R47H/+^ compared to SHAM WT littermates (*p*=.0307 by Tukey post-hoc test).

Overall, the results of the mechanical testing suggest that the presence of the TREM2 R47H variant in males partially protects pre-yield deficits but leads to reduced post-yield behavior after orchiectomy, whereas female mutants appear to be partially mechanically protected after gonadectomy.

### TREM2^R47H/+^ male mice are similarly affected by gonadectomy as WT mice in cancellous bone of the distal femur

**Figure 6:**
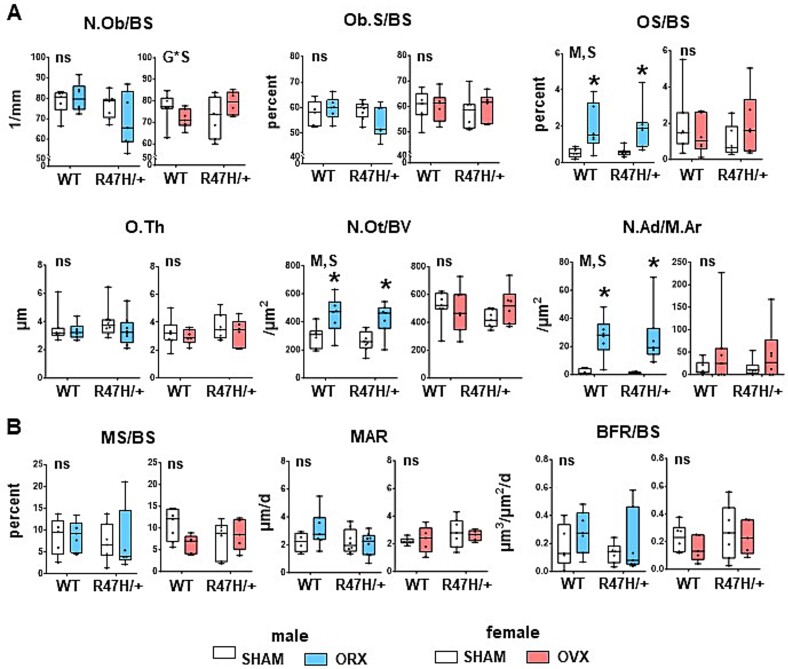
Gonadectomy leads to higher osteoid surface, osteocyte density, and marrow adipocyte abundance in male, independently of the expression of the TREM2 R47H variant. (A) Static and (B) dynamic histomorphometric parameters measured in the distal femur. Minimum to maximum values with median and IQR (25th to 75th percentile) are shown, and each dot corresponds to an individual sample. ^*^ = *p*<.05 vs corresponding SHAM, determined using linear regression models. M = overall model significant effect, S = surgery effect, G = genotype effect, and G^*^S = genotype × surgery interaction. *N* = 8-11/group. *N* = 5-7/group. Abbreviations: TREM2, triggering receptor expressed on myeloid cells 2; IQR, interquartile range.

Cancellous bone was drastically affected in orchiectomized mice independently of the genotype, with lower relative bone volume, trabecular thickness and number, and higher trabecular separation (Figure 5). It should be noted, however, that these differences do not explain the differential effect of surgery depending on sex and genotype observed at the BMD level (Figure 2). Changes in trabecular bone geometry were accompanied by increased osteoid volume, but no difference in other osteoblast-related measurements in cancellous bone of gonadectomized males of either genotype (Figure 6). Osteocyte density (including both alive and apoptotic osteocytes) was higher upon orchiectomy in mice of either genotype showing overall significance (M) and surgery effect (S), whereas it was not affected by either genotype or surgery in female mice (Figure 6A and Table S2). Similarly, adipocyte density in the bone marrow was higher in both WT and TREM2^R47H/+^ gonadectomized males, with overall significance (M) and surgery effect (S), compared to SHAM male mice. No differences were detected in dynamic histomorphometric parameters for either sex or genotype after surgery, with the exception of mineral apposition rate (MAR) (Figure 6B), which showed a *p* value of .05605 (Table S2) for the genotype × surgery interaction only in male mice.

On the other hand, the only difference detected for females was a slight but significantly lower trabecular thickness and higher trabecular separation induced by ovariectomy only in WT mice (Figure 5). There was also a significant genotype × surgery interaction for osteoblast numbers in females, but the post-hoc tests did not show differences between any 2 groups (Figure 6 and Table S2).

To further understand the consequences of gonadectomy in the context of the TREM2 R47H variant at the systemic level, selected chemokines were measured in serum collected 3 wk after surgery, using a multiplex array (Figure S3 and Table S2). Out of the 32 chemokines measured in the assay, we detected the presence of 9 with levels within the limits of detection. Of those, CCL5 was higher in TREM2^R47H/+^ orchiectomized mice, and showed a tendency toward higher values in WT ovariectomized mice (*p*=0.0558) (Figure S3 and Table S2). CCL11 (also known as eotaxin) showed a tendency toward lower values in WT mice (*p*=.05820, Table S2) and was significantly lower in orchiectomized TREM2^R47H/+^ mice, but it was not altered in serum from female mice. Surgery also affected CXCL1 levels in females, although none of the post-hoc tests showed differences between conditions. Although several changes were detected in chemokine levels, none of them explain the differences observed in the bone and skeletal muscle phenotypes. Yet, our data show that surgery and sex can alter the release of chemokines to the circulation, a subject that remains to be studied.

## Discussion

Previous studies from our group demonstrated that global expression of a copy of the R47H variant of the TREM2 gene, associated with increased risk of developing AD, results in reduced bone mass and strength, and altered skeletal muscle strength in a sex-dimorphic manner.^11^ This phenotype is found even in the absence of central neuropathology, suggesting direct effects of the TREM2 gene in bone. In particular, female TREM2^R47H/+^ mice exhibit reduced bone mass accrual and lower bone mass and strength at 13 mo of age (middle-aged mice). In the current study, we perform gonadectomy in adult 4-mo-old mice, in order to determine whether, as with aging, TREM2^R47H/+^ mice are more susceptible to the effects of sex steroid removal than the WT mice. However, we found an ameliorated effect of sex steroid removal in TREM2^R47H/+^ females on BMD and cortical bone strength, whereas the genotype had minimal effects on the effect of gonadectomy in males. This evidence provides further support to the notion that the mechanism of bone and muscle deterioration with aging differs from those of reduced sex steroid hormones.^27^

Our published study showed no differences between WT and TREM2^R47H/+^ mice in structural femoral bone parameters or in biomechanical strength in male and female mice, with the exception of a lower pre-yield displacement only in 4-mo-old female mice, and higher marrow area and decrease in relative bone volume observed in the mutant females at 13 mo of age.^11^ These previous findings contrast with the current report, in which we found that the SHAM-operated mutant female mice have higher relative cortical bone area, and lower ultimate force compared to WT littermate controls. Whether the inconsistency between the 2 studies is due to the different age of the mice (4- and 13- vs 5.5-mo-old), or the fact that in one experiment the mice were intact whereas they were SHAM-operated in the other, remains to be determined. Nevertheless, this evidence adds further support to the notion of accelerated skeletal aging in the presence of the TREM2 R47H variant only in female mice. We also found that by 20 mo of age, intact WT female mice exhibited a decline in bone parameters, yielding them no difference than TREM2^R47H/+^ littermates.^11^ These pieces of evidence suggest that the consequences of expression of the mutated TREM2 gene are highly dependent on the age of the mice studied. The molecular mechanism for the accelerated aging remains to be determined.

Gonadal sex hormones are master regulators of the energy metabolism in mice, and their deficiency after gonadectomy results in age- and sex-dependent changes on body weight and body composition. Particularly, Klappenbach et al. reported mice of both sexes reaching sexual maturity (2-mo-old) gain fat mass after gonadectomy, whereas body weight exhibited a sex dimorphic consequence of the surgery, with lower values in males and higher in females, 5 wk after the interventions.^28^ We reproduced these findings in our 4-mo-old WT mice in males and females at 3 and 6 wk after gonadectomy. On the other hand, while expression of the variant appeared to have minimal effects on BMD in orchiectomized mice, it may have also prevented the decrease in BMD 6-wk post-ovariectomy. Further studies will be needed to determine the molecular mechanism for this sexual dimorphic consequence of the mutated gene.

Although the difference was small, we found significantly lower trabecular thickness and higher spacing only in ovariectomized WT mice, whereas no changes in relative bone volume or trabecular number were seen in female mice of either genotype. Further, although in our previous study we found increased osteoclastogenesis when bone marrow cells from male TREM2^R47H/+^ mice were treated with M-CSF and sRANKL, no differences were detected in females with only a tendency toward reduced osteoclastogenesis when cells from mutant mice were used.^11^ In this previous study, we also reported that osteoclasts derived from female, but not male, TREM2^R47H/+^ mice appear to have reduced sensitivity to estrogen. Therefore, it is possible that in the female mutant mice, osteoclastic cells or their precursors are unsensitized to the removal of estrogens and, therefore, less affected by gonadectomy. This will be the subject of future studies.

In spite of the mild effect of the mutated gene on the consequences of gonadectomy in BMD, more in-depth analyses at the structural and biomechanical level showed that expression of the TREM2 R47H variant results in disparate response to gonadectomy compared to WT mice, and that the sex of the mice also influences the responses. Yet, very few of the measurements of cell number and activity were affected by surgery or genotype when analyzed 6-wk post-gonadectomy. It is possible that by analyzing the bones at the time of euthanasia we missed the times in which changes occurred at the cellular level. Consistent with this notion, the circulating levels of the marker of resorption were higher in females at the 3-wk, but not at the 6-wk time point. Further studies will be needed at an earlier time point to determine the cellular and molecular basis of the mutated TREM2-dependent differences at the tissue level.

In summary, our evidence shows that expression of the TREM2 R47H variant associated with increased risk of developing AD partially changes the outcomes of gonadectomy in the musculoskeletal system. In addition, the consequences of the mutant gene depend on the sex of the animals, with minimal effects on the deleterious effects of orchiectomy in the males and prevention of some of the effects of ovariectomy in females. Together with our previous study in intact mice,^11^ this study suggests that while gonadal hormones are necessary for the maintenance of the female skeleton in WT animals, estrogens can negatively affect bone and skeletal muscle in the presence of the TREM2 R47H variant in intact animals. Further, removal of the ovaries appears to partially prevent the negative effect of ovariectomy in TREM2^R47H/+^ mice. On the other hand, the TREM2 R47H variant does not alter the male skeleton up to 20 mo of age^11^ or its overall response to sex steroid removal in adult male mice (this study).

## Supplementary Material

Suppl_Fig_1_ziae144

Suppl_Fig_2_ziae144

Suppl_Fig_3_ziae144

Suppl_Table_1_ziae144

Suppl_Table_2_ziae144

## Data Availability

The data that support the findings of this study are available from the corresponding author, [L.I,P,], upon reasonable request.
