# Supplementary figures and images for: Sex hormone deficiency in male and female mice expressing the Alzheimer’s disease-associated risk-factor TREM2 R47H variant impacts the musculoskeletal system in a sex- and genotype-dependent manner
Source: JBMR Plus. 2024 Nov 13;9(1):ziae144. doi: 10.1093/jbmrpl/ziae144 (PMC11646090; doi:10.1093/jbmrpl/ziae144)

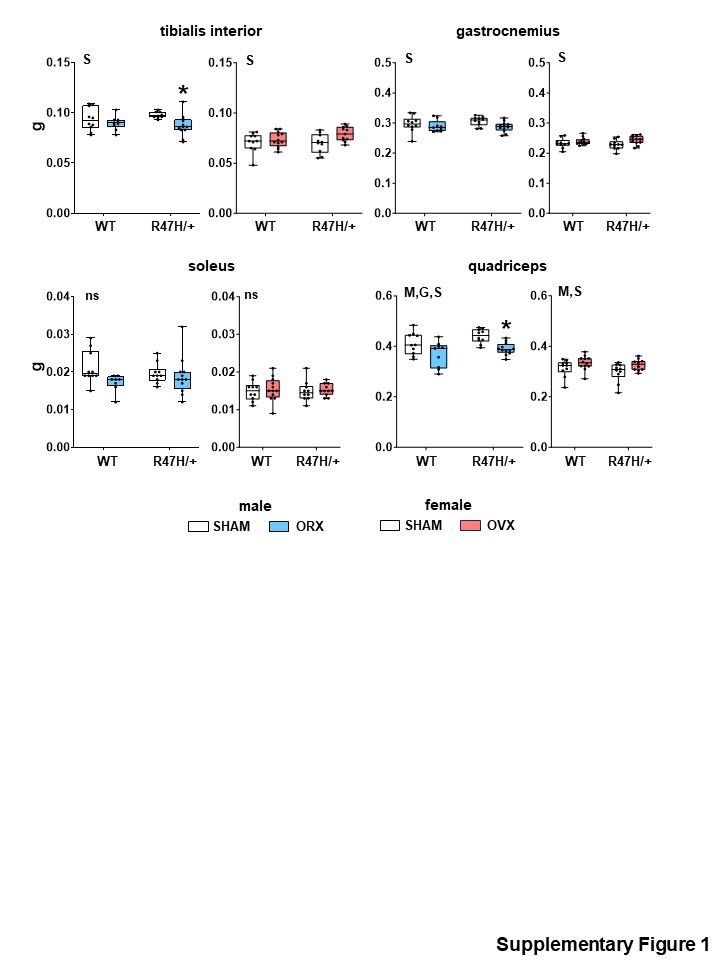

Supplement: Suppl_Fig_1_ziae144 [file suppl_fig_1_ziae144.jpeg]

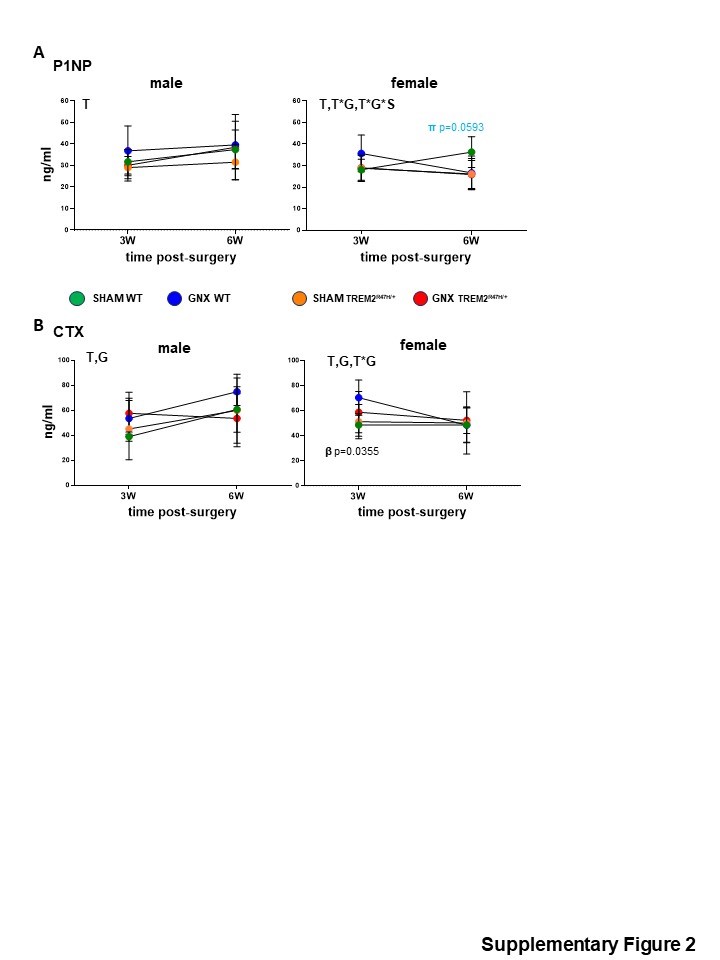

Supplement: Suppl_Fig_2_ziae144 [file suppl_fig_2_ziae144.jpeg]

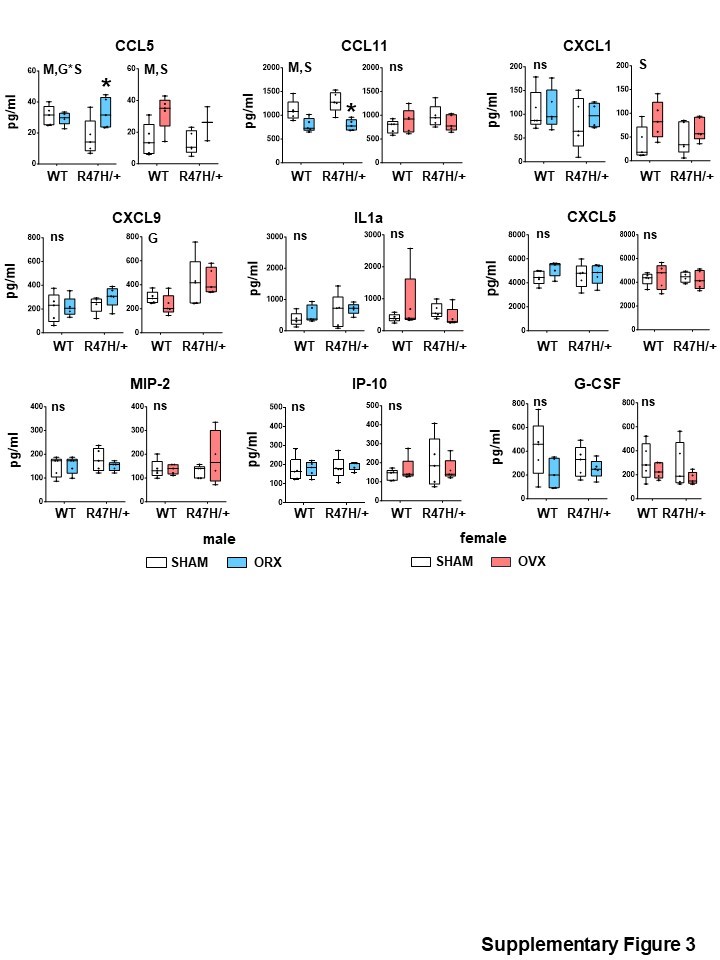

Supplement: Suppl_Fig_3_ziae144 [file suppl_fig_3_ziae144.jpeg]
