# Supplementary material for: Sex hormone deficiency in male and female mice expressing the Alzheimer’s disease-associated risk-factor TREM2 R47H variant impacts the musculoskeletal system in a sex- and genotype-dependent manner
Source: JBMR Plus. 2024 Nov 13;9(1):ziae144. doi: 10.1093/jbmrpl/ziae144 (PMC11646090; doi:10.1093/jbmrpl/ziae144)
Supplement: Suppl_Table_1_ziae144 [file suppl_table_1_ziae144.pdf]

Fig. 1

| outcome | Source | MALE P value | FEMALE P value |
|---|---|---|---|
| body weight | TIME POST-SURGERY | 0.0007 | <0.0001 |
| | GENOTYPE | <0.0001 | <0.0001 |
| | SURGERY | 0.0165 | 0.6508 |
| | TIME POST-SURGERY x GENOTYPE | 0.0006 | 0.0003 |
| | TIME POST-SURGERY x SURGERY | 0.9195 | 0.8833 |
| | GENOTYPE x SURGERY | 0.9240 | 0.0026 |
| | TIME POST-SURGERY x GENOTYPE x SURGERY | 0.6007 | 0.7614 |

| outcome | Source | P value | P value |
|---|---|---|---|
| lean mass | TIME POST-SURGERY | 0.1656 | <0.0001 |
| | GENOTYPE | 0.0014 | <0.0001 |
| | SURGERY | 0.0539 | 0.9139 |
| | TIME POST-SURGERY x GENOTYPE | 0.0025 | 0.0004 |
| | TIME POST-SURGERY x SURGERY | 0.9584 | 0.7106 |
| | GENOTYPE x SURGERY | 0.6404 | 0.0586 |
| | TIME POST-SURGERY x GENOTYPE x SURGERY | 0.9301 | 0.9130 |

| outcome | Source | P value | P value |
|---|---|---|---|
| fat mass | TIME POST-SURGERY | <0.0001 | <0.0001 |
| | GENOTYPE | <0.0001 | <0.0001 |
| | SURGERY | 0.0152 | 0.9521 |
| | TIME POST-SURGERY x GENOTYPE | 0.0886 | <0.0001 |
| | TIME POST-SURGERY x SURGERY | 0.8321 | 0.8666 |
| | GENOTYPE x SURGERY | 0.0162 | 0.1190 |
| | TIME POST-SURGERY x GENOTYPE x SURGERY | 0.7748 | 0.7193 |

| outcome | Source | P value | P value |
|---|---|---|---|
| percent lean mas | TIME POST-SURGERY | <0.0001 | <0.0001 |
| | GENOTYPE | 0.0213 | 0.0011 |
| | SURGERY | 0.0546 | 0.6872 |
| | TIME POST-SURGERY x GENOTYPE | 0.3268 | 0.0002 |
| | TIME POST-SURGERY x SURGERY | 0.6257 | 0.9232 |
| | GENOTYPE x SURGERY | 0.5058 | 0.0106 |
| | TIME POST-SURGERY x GENOTYPE x SURGERY | 0.2851 | 0.4727 |

| outcome | Source | P value | P value |
|---|---|---|---|
| percent fat mass | TIME POST-SURGERY | <0.0001 | <0.0001 |
| | GENOTYPE | 0.0067 | <0.0001 |
| | SURGERY | 0.0047 | 0.6145 |
| | TIME POST-SURGERY x GENOTYPE | 0.2767 | <0.0001 |
| | TIME POST-SURGERY x SURGERY | 0.8505 | 0.5881 |
| | GENOTYPE x SURGERY | 0.1184 | 0.4060 |
| | TIME POST-SURGERY x GENOTYPE x SURGERY | 0.8356 | 0.7648 |

| outcome | Source | P value | P value |
|---|---|---|---|

| | | | |
|---|---|---|---|
| total BMD | TIME POST-SURGERY | <0.0001 | 0.0001 |
| | GENOTYPE | <0.0001 | 0.003 |
| | SURGERY | 0.0579 | 0.1627 |
| | TIME POST-SURGERY x GENOTYPE | <0.0001 | 0.0011 |
| | TIME POST-SURGERY x SURGERY | 0.4008 | 0.5653 |
| | GENOTYPE x SURGERY | 0.6639 | 0.0029 |
| | TIME POST-SURGERY x GENOTYPE x SURGERY | 0.8692 | 0.6086 |

**Fig. 2**

| outcome | Source | P value | P value |
|---|---|---|---|
| femur BMD | TIME POST-SURGERY | <0.0001 | 0.0437 |
| | GENOTYPE | <0.0001 | 0.0039 |
| | SURGERY | 0.0880 | 0.2736 |
| | TIME POST-SURGERY x GENOTYPE | <0.0001 | 0.1214 |
| | TIME POST-SURGERY x SURGERY | 0.3175 | 0.9311 |
| | GENOTYPE x SURGERY | 0.2941 | 0.0008 |
| | TIME POST-SURGERY x GENOTYPE x SURGERY | 0.8762 | 0.8656 |

| outcome | Source | P value | P value |
|---|---|---|---|
| spine BMD | TIME POST-SURGERY | <0.0001 | <0.0001 |
| | GENOTYPE | 0.0089 | 0.0002 |
| | SURGERY | 0.4541 | 0.6414 |
| | TIME POST-SURGERY x GENOTYPE | 0.0002 | <0.0001 |
| | TIME POST-SURGERY x SURGERY | 0.5965 | 0.8549 |
| | GENOTYPE x SURGERY | 0.1385 | 0.0860 |
| | TIME POST-SURGERY x GENOTYPE x SURGERY | 0.9732 | 0.6175 |

| outcome | Source | P value | P value |
|---|---|---|---|
| P1NP | TIME POST-SURGERY | 0.0281 | 0.2756 |
| | GENOTYPE | 0.0810 | 0.7246 |
| | SURGERY | 0.0625 | 0.0071 |
| | TIME POST-SURGERY x GENOTYPE | 0.7338 | 0.0048 |
| | TIME POST-SURGERY x SURGERY | 0.7727 | 0.4156 |
| | GENOTYPE x SURGERY | 0.9302 | 0.7464 |
| | TIME POST-SURGERY x GENOTYPE x SURGERY | 0.3118 | 0.0075 |

**Suppl. Fig. 2**

| outcome | Source | P value | P value |
|---|---|---|---|
| CTX | TIME POST-SURGERY | 0.0026 | 0.0331 |
| | GENOTYPE | 0.0468 | 0.0229 |
| | SURGERY | 0.4956 | 0.7916 |
| | TIME POST-SURGERY x GENOTYPE | 0.2659 | 0.0448 |
| | TIME POST-SURGERY x SURGERY | 0.0644 | 0.2866 |
| | GENOTYPE x SURGERY | 0.1971 | 0.3776 |
| | TIME POST-SURGERY x GENOTYPE x SURGERY | 0.2845 | 0.2242 |
