# Supplementary material for: Sex hormone deficiency in male and female mice expressing the Alzheimer’s disease-associated risk-factor TREM2 R47H variant impacts the musculoskeletal system in a sex- and genotype-dependent manner
Source: JBMR Plus. 2024 Nov 13;9(1):ziae144. doi: 10.1093/jbmrpl/ziae144 (PMC11646090; doi:10.1093/jbmrpl/ziae144)
Supplement: Suppl_Table_2_ziae144 [file suppl_table_2_ziae144.pdf]

	outcome	Source	MALE P value	FEMALE P value
Fig. 3A	B.Ar/T.Ar	Model	0.1272	0.0281
		Genotype	0.7292	0.0036
		Surgery	0.0533	0.7032
		Genotype*Surgery	0.1475	0.4582
		WTGNX vs WTSHAM	0.0868	>0.9999
		R47H/+GNX vs R47H/+SHAM	>0.9999	>0.9999
		R47H/+SHAM vs WTSHAM	0.7712	0.0482
		R47H/+GNX vs WTGNX	1.0000	0.3710
	Ct.Th	Model	0.1647	0.4292
		Genotype	0.7297	0.1273
		Surgery	0.0346	0.9538
		Genotype*Surgery	0.3590	0.4668
		WTGNX vs WTSHAM	0.1667	>0.9999
		R47H/+GNX vs R47H/+SHAM	>0.9999	>0.9999
		R47H/+SHAM vs WTSHAM	>0.9999	0.4996
		R47H/+GNX vs WTGNX	>0.9999	>0.9999
	Ma.Ar	Model	0.1435	0.0129
		Genotype	0.4422	0.0014
		Surgery	0.0538	0.5405
		Genotype*Surgery	0.2407	0.8325
		WTGNX vs WTSHAM	0.1520	>0.9999
		R47H/+GNX vs R47H/+SHAM	>0.9999	>0.9999
		R47H/+SHAM vs WTSHAM	0.6804	0.0678
		R47H/+GNX vs WTGNX	>0.9999	0.0933
	Imax	Model	0.1502	0.0323
		Genotype	0.1789	0.0054
		Surgery	0.3624	0.4170
		Genotype*Surgery	0.0902	0.7845
		WTGNX vs WTSHAM	0.3210	>0.9999
		R47H/+GNX vs R47H/+SHAM	>0.9999	>0.9999
		R47H/+SHAM vs WTSHAM	0.1334	0.3008
		R47H/+GNX vs WTGNX	>0.9999	0.0930
	TMD	Model	0.0031	0.2497
		Genotype	0.5628	0.5595
		Surgery	0.0003	0.1363
		Genotype*Surgery	0.7447	0.2449
		WTGNX vs WTSHAM	0.0218	0.2436
		R47H/+GNX vs R47H/+SHAM	0.0356	>0.9999
		R47H/+SHAM vs WTSHAM	>0.9999	>0.9999
		R47H/+GNX vs WTGNX	>0.9999	0.8096
	[illegible]	Model	0.4606	0.5297
		Genotype	0.1993	0.2294
		Surgery	0.5783	0.5371
		Genotype*Surgery	0.3037	0.6426

Figure	outcome	Source	P value	P value
	Ps.MS/BS	WTGNX vs WTSHAM	>0.9999	>0.9999
		R47H/+GNX vs R47H/+SHAM	>0.9999	>0.9999
		R47H/+SHAM vs WTSHAM	0.5125	>0.9999
		R47H/+GNX vs WTGNX	>0.9999	>0.9999

Figure	outcome	Source	P value	P value
Fig. 3B	Ps.MAR	Model	0.7996	0.0263
		Genotype	0.5144	0.9347
		Surgery	0.7229	0.0310
		Genotype*Surgery	0.5064	0.0431
		WTGNX vs WTSHAM	>0.9999	0.0111
		R47H/+GNX vs R47H/+SHAM	>0.9999	>0.9999
		R47H/+SHAM vs WTSHAM	>0.9999	0.4881
		R47H/+GNX vs WTGNX	>0.9999	0.6901

Figure	outcome	Source	P value	P value
Fig. 3B	Ps.BFR/BS	Model	0.0653	0.0577
		Genotype	0.2803	0.9307
		Surgery	0.0535	0.0206
		Genotype*Surgery	0.1621	0.1616
		WTGNX vs WTSHAM	>0.9999	0.0347
		R47H/+GNX vs R47H/+SHAM	0.0791	>0.9999
		R47H/+SHAM vs WTSHAM	0.3195	>0.9999
		R47H/+GNX vs WTGNX	>0.9999	>0.9999

Figure	outcome	Source	P value	P value
Fig. 3C	Ec.MS/BS	Model	0.6952	0.7675
		Genotype	0.6837	0.6865
		Surgery	0.4776	0.7656
		Genotype*Surgery	0.4206	0.3274
		WTGNX vs WTSHAM	>0.9999	>0.9999
		R47H/+GNX vs R47H/+SHAM	>0.9999	>0.9999
		R47H/+SHAM vs WTSHAM	>0.9999	>0.9999
		R47H/+GNX vs WTGNX	>0.9999	>0.9999

Figure	outcome	Source	P value	P value
Fig. 3C	Ec.MAR	Model	0.6327	0.8370
		Genotype	0.2455	0.7826
		Surgery	0.6231	0.4638
		Genotype*Surgery	0.9022	0.5669
		WTGNX vs WTSHAM	>0.9999	>0.9999
		R47H/+GNX vs R47H/+SHAM	>0.9999	>0.9999
		R47H/+SHAM vs WTSHAM	>0.9999	>0.9999
		R47H/+GNX vs WTGNX	>0.9999	>0.9999

Figure	outcome	Source	P value	P value
Fig. 3C	Ec.BFR/BS	Model	0.6852	0.6001
		Genotype	0.5675	0.2745
		Surgery	0.4070	0.8689
		Genotype*Surgery	0.5531	0.3817
		WTGNX vs WTSHAM	>0.9999	>0.9999
		R47H/+GNX vs R47H/+SHAM	>0.9999	>0.9999
		R47H/+SHAM vs WTSHAM	>0.9999	>0.9999
		R47H/+GNX vs WTGNX	>0.9999	0.7254

Fig. 3D

outcome	Source	P value	P value
N.EcOC/EcS	Model	0.3263	0.4792
	Genotype	0.2676	0.4566
	Surgery	0.1742	0.4699
	Genotype*Surgery	0.6599	0.2652
	WTGNX vs WTSHAM	>0.9999	0.6907
	R47H/+GNX vs R47H/+SHAM	0.7017	>0.9999
	R47H/+SHAM vs WTSHAM	>0.9999	>0.9999
	R47H/+GNX vs WTGNX	>0.9999	0.8975

outcome	Source	P value	P value
Ec.OcS/EcS	Model	0.2478	0.6353
	Genotype	0.1845	0.2643
	Surgery	0.2543	0.8426
	Genotype*Surgery	0.3632	0.4846
	WTGNX vs WTSHAM	>0.9999	>0.9999
	R47H/+GNX vs R47H/+SHAM	0.5027	>0.9999
	R47H/+SHAM vs WTSHAM	>0.9999	>0.9999
	R47H/+GNX vs WTGNX	0.4695	0.9630

outcome	Source	P value	P value
yield force	Model	0.0000	0.0014
	Genotype	0.8735	0.4762
	Surgery	0.0000	0.0004
	Genotype*Surgery	0.4184	0.0769
	WTGNX vs WTSHAM	0.0001	0.0013
	R47H/+GNX vs R47H/+SHAM	0.0014	0.5200
	R47H/+SHAM vs WTSHAM	>0.9999	0.3496
	R47H/+GNX vs WTGNX	>0.9999	>0.9999

outcome	Source	P value	P value
ultimate force	Model	0.0000	0.0007
	Genotype	0.9799	0.0518
	Surgery	0.0000	0.0010
	Genotype*Surgery	0.6205	0.0424
	WTGNX vs WTSHAM	0.0001	0.0016
	R47H/+GNX vs R47H/+SHAM	0.0000	>0.9999
	R47H/+SHAM vs WTSHAM	>0.9999	0.0296
	R47H/+GNX vs WTGNX	>0.9999	>0.9999

outcome	Source	P value	P value
stiffness	Model	0.0000	0.0008
	Genotype	0.7868	0.0512
	Surgery	0.0000	0.0010
	Genotype*Surgery	0.4067	0.0695
	WTGNX vs WTSHAM	0.0002	0.0024
	R47H/+GNX vs R47H/+SHAM	0.0000	0.8705
	R47H/+SHAM vs WTSHAM	>0.9999	0.0439
	R47H/+GNX vs WTGNX	>0.9999	>0.9999

outcome	Source	P value	P value
work to yield	Model	0.0012	0.0093
	Genotype	0.8956	0.8895
	Surgery	0.0001	0.0019
	Genotype*Surgery	0.1823	0.1293

work to yield	WTGNX vs WTSHAM	0.0016	0.0070
	R47H/+GNX vs R47H/+SHAM	0.1629	0.7861
	R47H/+SHAM vs WTSHAM	>0.9999	0.9959
	R47H/+GNX vs WTGNX	>0.9999	>0.9999

outcome	Source	P value	P value
post yield work	Model	0.0208	0.3130
	Genotype	0.6476	0.3669
	Surgery	0.0221	0.5640
	Genotype*Surgery	0.0416	0.1119
	WTGNX vs WTSHAM	>0.9999	>0.9999
	R47H/+GNX vs R47H/+SHAM	0.0095	0.4739
	R47H/+SHAM vs WTSHAM	0.2800	0.3491
	R47H/+GNX vs WTGNX	>0.9999	>0.9999

outcome	Source	P value	P value
total work	Model	0.0150	0.2992
	Genotype	0.6574	0.3689
	Surgery	0.0115	0.8093
	Genotype*Surgery	0.0533	0.0889
	WTGNX vs WTSHAM	>0.9999	>0.9999
	R47H/+GNX vs R47H/+SHAM	0.0070	0.6322
	R47H/+SHAM vs WTSHAM	0.3379	0.2976
	R47H/+GNX vs WTGNX	>0.9999	>0.9999

outcome	Source	P value	P value
yield stress	Model	0.0492	0.0035
	Genotype	0.9821	0.7027
	Surgery	0.0073	0.0007
	Genotype*Surgery	0.3833	0.1193
	WTGNX vs WTSHAM	0.0576	0.0032
	R47H/+GNX vs R47H/+SHAM	0.6526	0.5161
	R47H/+SHAM vs WTSHAM	>0.9999	0.7161
	R47H/+GNX vs WTGNX	>0.9999	>0.9999

Fig. 4

outcome	Source	P value	P value
ultimate stress	Model	0.0793	0.0018
	Genotype	0.8746	0.1369
	Surgery	0.0104	0.0015
	Genotype*Surgery	0.9538	0.0554
	WTGNX vs WTSHAM	0.2581	0.0026
	R47H/+GNX vs R47H/+SHAM	0.2509	>0.9999
	R47H/+SHAM vs WTSHAM	>0.9999	0.0834
	R47H/+GNX vs WTGNX	>0.9999	>0.9999

outcome	Source	P value	P value
modulus	Model	0.1463	0.0306
	Genotype	0.6992	0.3030
	Surgery	0.0246	0.0247
	Genotype*Surgery	0.9035	0.0735
	WTGNX vs WTSHAM	0.5346	0.0261
	R47H/+GNX vs R47H/+SHAM	0.3269	>0.9999
	R47H/+SHAM vs WTSHAM	>0.9999	0.2142
	R47H/+GNX vs WTGNX	>0.9999	>0.9999

outcome	Source	P value	P value
resilience	Model	0.0343	0.0137
	Genotype	0.8221	0.9654
	Surgery	0.0068	0.0018
	Genotype*Surgery	0.1994	0.3335
	WTGNX vs WTSHAM	0.0264	0.0187
	R47H/+GNX vs R47H/+SHAM	>0.9999	0.3519
	R47H/+SHAM vs WTSHAM	>0.9999	>0.9999
	R47H/+GNX vs WTGNX	>0.9999	>0.9999

outcome	Source	P value	P value
toughness	Model	0.0342	0.4682
	Genotype	0.5077	0.3603
	Surgery	0.0411	0.7915
	Genotype*Surgery	0.0500	0.1902
	WTGNX vs WTSHAM	>0.9999	>0.9999
	R47H/+GNX vs R47H/+SHAM	0.0191	>0.9999
	R47H/+SHAM vs WTSHAM	0.2326	0.5069
	R47H/+GNX vs WTGNX	>0.9999	>0.9999

outcome	Source	P value	P value
failure force	Model	0.0912	0.0774
	Genotype	0.6381	0.9687
	Surgery	0.0322	0.0116
	Genotype*Surgery	0.1435	0.5920
	WTGNX vs WTSHAM	0.0656	0.1132
	R47H/+GNX vs R47H/+SHAM	>0.9999	0.5924
	R47H/+SHAM vs WTSHAM	0.6809	>0.9999
	R47H/+GNX vs WTGNX	>0.9999	>0.9999

outcome	Source	P value	P value
ultimate strain	Model	0.2174	0.4169
	Genotype	0.1081	0.3722
	Surgery	0.2416	0.2010
	Genotype*Surgery	0.6453	0.5588
	WTGNX vs WTSHAM	>0.9999	0.7564
	R47H/+GNX vs R47H/+SHAM	0.9058	>0.9999
	R47H/+SHAM vs WTSHAM	>0.9999	>0.9999
	R47H/+GNX vs WTGNX	0.5835	>0.9999

outcome	Source	P value	P value
displacement to yield	Model	0.2870	0.8194
	Genotype	0.6603	0.7834
	Surgery	0.9909	0.4574
	Genotype*Surgery	0.0621	0.5908
	WTGNX vs WTSHAM	0.7971	>0.9999
	R47H/+GNX vs R47H/+SHAM	0.6539	>0.9999
	R47H/+SHAM vs WTSHAM	>0.9999	>0.9999
	R47H/+GNX vs WTGNX	0.4166	>0.9999

outcome	Source	P value	P value
cancellous BV/TV	Model	0.0000	0.2108
	Genotype	0.6467	0.2255
	Surgery	0.0000	0.9536
	Genotype*Surgery	0.7631	0.0767

Fig. 5

cancellous BV/TV	WTGNX vs WTSHAM	0.0000	0.9048
	R47H/+GNX vs R47H/+SHAM	0.0000	0.7432
	R47H/+SHAM vs WTSHAM	>0.9999	0.1598
	R47H/+GNX vs WTGNX	>0.9999	>0.9999

outcome	Source	P value	P value
Tb.Th	Model	0.0000	0.0034
	Genotype	0.7206	0.6960
	Surgery	0.0000	0.0004
	Genotype*Surgery	0.7251	0.2650
	WTGNX vs WTSHAM	0.0027	0.0051
	R47H/+GNX vs R47H/+SHAM	0.0003	0.2243
	R47H/+SHAM vs WTSHAM	>0.9999	>0.9999
	R47H/+GNX vs WTGNX	>0.9999	>0.9999

outcome	Source	P value	P value
Tb.N	Model	0.0000	0.1114
	Genotype	0.5456	0.1730
	Surgery	0.0000	0.1726
	Genotype*Surgery	0.8707	0.1253
	WTGNX vs WTSHAM	0.0000	>0.9999
	R47H/+GNX vs R47H/+SHAM	0.0000	0.1632
	R47H/+SHAM vs WTSHAM	>0.9999	0.1850
	R47H/+GNX vs WTGNX	>0.9999	>0.9999

outcome	Source	P value	P value
Tb.Sp	Model	0.0000	0.0563
	Genotype	0.5221	0.6871
	Surgery	0.0000	0.0286
	Genotype*Surgery	0.6990	0.0906
	WTGNX vs WTSHAM	0.0000	0.0324
	R47H/+GNX vs R47H/+SHAM	0.0000	>0.9999
	R47H/+SHAM vs WTSHAM	>0.9999	>0.9999
	R47H/+GNX vs WTGNX	>0.9999	0.5315

outcome	Source	P value	P value
vBMD	Model	0.2043	0.0486
	Genotype	0.5894	0.9649
	Surgery	0.0389	0.0061
	Genotype*Surgery	0.8546	0.6984
	WTGNX vs WTSHAM	0.4845	0.0997
	R47H/+GNX vs R47H/+SHAM	0.6350	0.3270
	R47H/+SHAM vs WTSHAM	>0.9999	>0.9999
	R47H/+GNX vs WTGNX	>0.9999	>0.9999

outcome	Source	P value	P value
N.Ob/BS	Model	0.1309	0.1480
	Genotype	0.0623	0.6433
	Surgery	0.4637	0.7777
	Genotype*Surgery	0.1681	0.0254
	WTGNX vs WTSHAM	>0.9999	0.5698
	R47H/+GNX vs R47H/+SHAM	0.4997	0.3056
	R47H/+SHAM vs WTSHAM	>0.9999	0.7158
	R47H/+GNX vs WTGNX	0.1202	0.2379

Fig. 6A

outcome	Source	P value	P value
Ob.S/BS	Model	0.1814	0.9072
	Genotype	0.2741	0.6321
	Surgery	0.5291	0.6871
	Genotype*Surgery	0.0663	0.7250
	WTGNX vs WTSHAM	>0.9999	>0.9999
	R47H/+GNX vs R47H/+SHAM	0.2885	>0.9999
	R47H/+SHAM vs WTSHAM	>0.9999	>0.9999
	R47H/+GNX vs WTGNX	0.1942	>0.9999

outcome	Source	P value	P value
OS/BS	Model	0.0030	0.5654
	Genotype	0.9037	0.7747
	Surgery	0.0003	0.7676
	Genotype*Surgery	0.9764	0.1812
	WTGNX vs WTSHAM	0.0318	>0.9999
	R47H/+GNX vs R47H/+SHAM	0.0171	>0.9999
	R47H/+SHAM vs WTSHAM	>0.9999	0.9657
	R47H/+GNX vs WTGNX	>0.9999	>0.9999

outcome	Source	P value	P value
O.Th	Model	0.6193	0.5433
	Genotype	0.5596	0.3016
	Surgery	0.2883	0.3330
	Genotype*Surgery	0.6657	0.9051
	WTGNX vs WTSHAM	>0.9999	>0.9999
	R47H/+GNX vs R47H/+SHAM	>0.9999	>0.9999
	R47H/+SHAM vs WTSHAM	>0.9999	>0.9999
	R47H/+GNX vs WTGNX	>0.9999	>0.9999

outcome	Source	P_value	P_value
N.Ot/BV	Model	0.0033	0.4959
	Genotype	0.3769	0.4955
	Surgery	0.0004	0.4507
	Genotype*Surgery	0.9733	0.2570
	WTGNX vs WTSHAM	0.0388	>0.9999
	R47H/+GNX vs R47H/+SHAM	0.0210	0.7760
	R47H/+SHAM vs WTSHAM	>0.9999	0.7750
	R47H/+GNX vs WTGNX	>0.9999	>0.9999

outcome	Source	P_value	P_value
N.Ad/M.Ar	Model	0.0006	0.5038
	Genotype	0.9192	0.9110
	Surgery	0.0000	0.1404
	Genotype*Surgery	0.9886	0.8819
	WTGNX vs WTSHAM	0.0068	0.9551
	R47H/+GNX vs R47H/+SHAM	0.0065	>0.9999
	R47H/+SHAM vs WTSHAM	>0.9999	>0.9999
	R47H/+GNX vs WTGNX	>0.9999	>0.9999

outcome	Source	P value	P value
MS/BS	Model	0.9272	0.4543
	Genotype	0.5458	0.7644
	Surgery	0.8261	0.1752
	Genotype*Surgery	0.9392	0.4048

	MS/BS	WTGNX vs WTSHAM	>0.9999	0.5050
		R47H/+GNX vs R47H/+SHAM	>0.9999	>0.9999
		R47H/+SHAM vs WTSHAM	>0.9999	>0.9999
		R47H/+GNX vs WTGNX	>0.9999	>0.9999

	outcome	Source	P value	P value
Fig. 6B	MAR	Model	0.1086	0.8708
		Genotype	0.4062	0.8253
		Surgery	0.1509	0.4652
		Genotype*Surgery	0.0561	0.7294
		WTGNX vs WTSHAM	0.1230	>0.9999
		R47H/+GNX vs R47H/+SHAM	>0.9999	>0.9999
		R47H/+SHAM vs WTSHAM	>0.9999	>0.9999
		R47H/+GNX vs WTGNX	0.1773	>0.9999

	outcome	Source	P value	P value
Fig. 6B	BFR/BS	Model	0.8772	0.5203
		Genotype	0.9729	0.9488
		Surgery	0.8477	0.2156
		Genotype*Surgery	0.4250	0.4154
		WTGNX vs WTSHAM	>0.9999	0.6049
		R47H/+GNX vs R47H/+SHAM	>0.9999	>0.9999
		R47H/+SHAM vs WTSHAM	>0.9999	>0.9999
		R47H/+GNX vs WTGNX	>0.9999	>0.9999

	outcome	Source	P value	P value
Supp. Fig. 1A	tibialis interior	Model	0.0640	0.0735
		Genotype	0.7619	0.3975
		Surgery	0.0166	0.0269
		Genotype*Surgery	0.2512	0.2239
		WTGNX vs WTSHAM	>0.9999	>0.9999
		R47H/+GNX vs R47H/+SHAM	0.0468	0.0710
		R47H/+SHAM vs WTSHAM	>0.9999	>0.9999
		R47H/+GNX vs WTGNX	>0.9999	0.5340

	outcome	Source	P value	P value
Supp. Fig. 1A	gastrocnemius	Model	0.1632	0.1042
		Genotype	0.5915	0.8715
		Surgery	0.0471	0.0229
		Genotype*Surgery	0.3851	0.3141
		WTGNX vs WTSHAM	>0.9999	>0.9999
		R47H/+GNX vs R47H/+SHAM	0.1523	0.0907
		R47H/+SHAM vs WTSHAM	>0.9999	>0.9999
		R47H/+GNX vs WTGNX	>0.9999	>0.9999

	outcome	Source	P value	P value
Supp. Fig. 1A	soleus	Model	0.1895	0.9521
		Genotype	0.9658	0.9280
		Surgery	0.0615	0.5773
		Genotype*Surgery	0.1955	0.9280
		WTGNX vs WTSHAM	0.1407	>0.9999
		R47H/+GNX vs R47H/+SHAM	>0.9999	>0.9999
		R47H/+SHAM vs WTSHAM	>0.9999	>0.9999
		R47H/+GNX vs WTGNX	>0.9999	>0.9999

outcome	Source	P value	P value
quadriceps	Model	0.0010	0.0434
	Genotype	0.0279	0.2070
	Surgery	0.0003	0.0115
	Genotype*Surgery	0.7476	0.6595
	WTGNX vs WTSHAM	0.0699	0.4907
	R47H/+GNX vs R47H/+SHAM	0.0131	0.1453
	R47H/+SHAM vs WTSHAM	0.2983	0.9785
	R47H/+GNX vs WTGNX	0.6803	>0.9999

outcome	Source	P value	P value
CCL5	Model	0.0410	0.0271
	Genotype	0.1960	0.3651
	Surgery	0.0879	0.0075
	Genotype*Surgery	0.0352	0.5858
	WTGNX vs WTSHAM	>0.9999	0.0558
	R47H/+GNX vs R47H/+SHAM	0.0407	0.4493
	R47H/+SHAM vs WTSHAM	0.0803	>0.9999
	R47H/+GNX vs WTGNX	>0.9999	>0.9999

outcome	Source	P value	P value
CCL11	Model	0.0009	0.4040
	Genotype	0.2633	0.3496
	Surgery	0.0001	0.8997
	Genotype*Surgery	0.2745	0.1612
	WTGNX vs WTSHAM	0.0582	>0.9999
	R47H/+GNX vs R47H/+SHAM	0.0020	>0.9999
	R47H/+SHAM vs WTSHAM	0.4992	0.4188
	R47H/+GNX vs WTGNX	>0.9999	>0.9999

outcome	Source	P value	P value
CXCL1	Model	0.6708	0.1496
	Genotype	0.3229	0.7620
	Surgery	0.5899	0.0387
	Genotype*Surgery	0.7148	0.3473
	WTGNX vs WTSHAM	>0.9999	0.1475
	R47H/+GNX vs R47H/+SHAM	>0.9999	>0.9999
	R47H/+SHAM vs WTSHAM	>0.9999	>0.9999
	R47H/+GNX vs WTGNX	>0.9999	>0.9999

outcome	Source	P value	P value
CXCL9	Model	0.4007	0.0675
	Genotype	0.1967	0.0117
	Surgery	0.3861	0.6723
	Genotype*Surgery	0.4821	0.5367
	WTGNX vs WTSHAM	>0.9999	>0.9999
	R47H/+GNX vs R47H/+SHAM	>0.9999	>0.9999
	R47H/+SHAM vs WTSHAM	>0.9999	0.5472
	R47H/+GNX vs WTGNX	0.6529	0.1027

outcome	Source	P value	P value
IL1a	Model	0.4571	0.4890
	Genotype	0.1807	0.6524
	Surgery	0.4307	0.5795
	Genotype*Surgery	0.7290	0.1759

Supp. Fig. 3A

IL1a	WTGNX vs WTSHAM	>0.9999	0.7209
	R47H/+GNX vs R47H/+SHAM	>0.9999	>0.9999
	R47H/+SHAM vs WTSHAM	0.9329	>0.9999
	R47H/+GNX vs WTGNX	>0.9999	0.8139

outcome	Source	P value	P value
CXCL5	Model	0.5204	0.9437
	Genotype	0.6614	0.8650
	Surgery	0.2480	0.9502
	Genotype*Surgery	0.4106	0.5734
	WTGNX vs WTSHAM	0.6707	>0.9999
	R47H/+GNX vs R47H/+SHAM	>0.9999	>0.9999
	R47H/+SHAM vs WTSHAM	>0.9999	>0.9999
	R47H/+GNX vs WTGNX	>0.9999	>0.9999

outcome	Source	P value	P value
MIP-2	Model	0.6135	0.4845
	Genotype	0.4881	0.5038
	Surgery	0.5478	0.3019
	Genotype*Surgery	0.3387	0.2828
	WTGNX vs WTSHAM	>0.9999	>0.9999
	R47H/+GNX vs R47H/+SHAM	>0.9999	0.6172
	R47H/+SHAM vs WTSHAM	0.9915	>0.9999
	R47H/+GNX vs WTGNX	>0.9999	0.9416

outcome	Source	P value	P value
IP-10	Model	0.9282	0.6501
	Genotype	0.5905	0.3720
	Surgery	0.7239	0.8781
	Genotype*Surgery	0.8923	0.3822
	WTGNX vs WTSHAM	>0.9999	>0.9999
	R47H/+GNX vs R47H/+SHAM	>0.9999	>0.9999
	R47H/+SHAM vs WTSHAM	>0.9999	0.8686
	R47H/+GNX vs WTGNX	>0.9999	>0.9999

outcome	Source	P value	P value
G-CSF	Model	0.1994	0.3549
	Genotype	0.6225	0.4038
	Surgery	0.0678	0.1217
	Genotype*Surgery	0.3042	0.7808
	WTGNX vs WTSHAM	0.1941	>0.9999
	R47H/+GNX vs R47H/+SHAM	>0.9999	0.7762
	R47H/+SHAM vs WTSHAM	>0.9999	>0.9999
	R47H/+GNX vs WTGNX	>0.9999	>0.9999
